# Analysis of Transcription Factors Key for Mouse Pancreatic Development Establishes *NKX2-2* and *MNX1* Mutations as Causes of Neonatal Diabetes in Man

**DOI:** 10.1016/j.cmet.2013.11.021

**Published:** 2014-01-07

**Authors:** Sarah E. Flanagan, Elisa De Franco, Hana Lango Allen, Michele Zerah, Majedah M. Abdul-Rasoul, Julie A. Edge, Helen Stewart, Elham Alamiri, Khalid Hussain, Sam Wallis, Liat de Vries, Oscar Rubio-Cabezas, Jayne A.L. Houghton, Emma L. Edghill, Ann-Marie Patch, Sian Ellard, Andrew T. Hattersley

**Affiliations:** 1 Institute of Biomedical and Clinical Science, University of Exeter Medical School, Exeter EX2 5DW, UK; 2 Presbyterian Medical Group, Albuquerque, NM 87106, USA; 3 Faculty of Medicine, Department of Pediatrics, Kuwait University, Safat 13110, Kuwait; 4 Oxford Children’s Hospital, Headington, Oxford OX3 9DU, UK; 5 Department of Clinical Genetics, Oxford University Hospitals NHS Trust, Oxford OX3 7LE, UK; 6 Al Qassimi Hospital, Sharjah 3500, United Arab Emirates; 7 London Centre for Paediatric Endocrinology and Metabolism, Great Ormond Street Hospital for Children NHS Trust, and The Institute of Child Health, University College London, London WC1N 1EH, UK; 8 Neonatal Unit, Bradford Royal Infirmary, Bradford BD9 6RJ, UK; 9 Institute of Endocrinology and Diabetes, Schneider Children’s Medical Center of Israel, PetahTikva, and Sackler Faculty of Medicine, Tel Aviv University, Tel Aviv 49202, Israel; 10 Department of Paediatric Endocrinology, Hospital Infantil Niño Jesús, Madrid 28009, Spain

## Abstract

Understanding transcriptional regulation of pancreatic development is required to advance current efforts in developing beta cell replacement therapies for patients with diabetes. Current knowledge of key transcriptional regulators has predominantly come from mouse studies, with rare, naturally occurring mutations establishing their relevance in man. This study used a combination of homozygosity analysis and Sanger sequencing in 37 consanguineous patients with permanent neonatal diabetes to search for homozygous mutations in 29 transcription factor genes important for murine pancreatic development. We identified homozygous mutations in 7 different genes in 11 unrelated patients and show that *NKX2*-2 and *MNX1* are etiological genes for neonatal diabetes, thus confirming their key role in development of the human pancreas. The similar phenotype of the patients with recessive mutations and mice with inactivation of a transcription factor gene support there being common steps critical for pancreatic development and validate the use of rodent models for beta cell development.

## Highlights

• Homozygous mutations in seven pancreatic transcription factors cause neonatal diabetes
• Homozygous *NKX2-2* and *MNX1* mutations were found in five patients
• Confirms NKX2-2 and MNX1 are critical for murine and human pancreas development
• Similar phenotypes in mouse and man validate models for beta cell development

## Graphical Abstract

**Figure:**
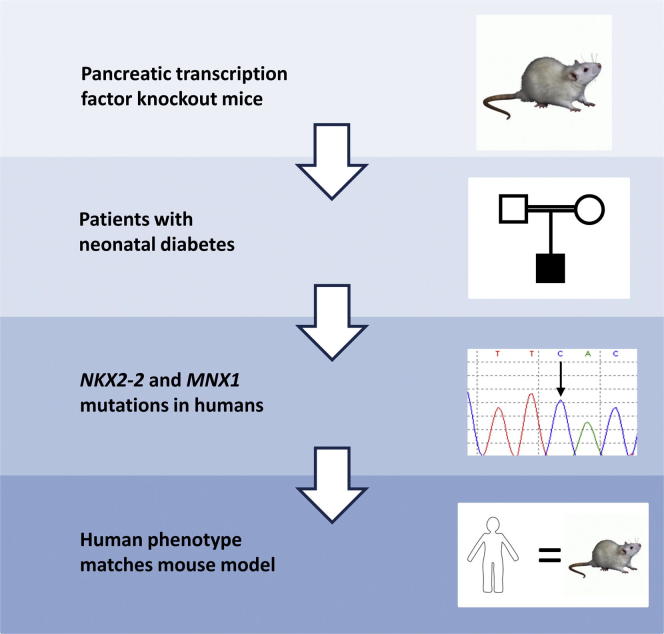

## Teaser

Analyzing a large collection of consanguineous patients with permanent neonatal diabetes, Flanagan et al. performed a comprehensive search for recessive mutations in transcription factors known to be critical for mouse pancreatic development. They identify seven pancreatic transcription factors that cause neonatal diabetes, including NKX2-2 and MNX1.

## Introduction

Knowledge on transcriptional regulators of pancreatic development and beta cell differentiation is essential for the development of beta cell replacement therapies for diabetes (Kroon et al., 2008, Zhou et al., 2008). While major work has been undertaken in mice, the extent to which murine pancreatic development is a good model for man is not fully understood.

Finding inactivating mutations in patients with diabetes can establish the role of a gene in human pancreatic development and function. Comparing the human phenotype with mouse models for the same gene is important, as it validates that the gene function is similar in man and mouse. Concordant phenotypes between mouse models with biallelic gene inactivation and humans with biallelic mutations suggest similar function of the gene product in rodents and man. For example, biallelic *GCK* mutations in humans cause permanent neonatal diabetes (PNDM), and mice lacking both copies of the gene have severe hyperglycemia at birth (Njølstad et al., 2001, Terauchi et al., 1995). Both patients with recessive *EIF2AK3* mutations and the *Perk* null mouse (*Eif2ak3*^−/−^) develop diabetes due to destruction of beta cells as a result of endoplasmic reticulum stress (Delépine et al., 2000, Harding et al., 2001, Zhang et al., 2002)

The correlation in phenotype between knockout mice and humans with recessive mutations is not always evident. Biallelic loss-of-function *ABCC8* mutations are the most common known cause of hyperinsulinemic hypoglycemia in humans, whereas *ABCC8* null mice maintain euglycemia (Shiota et al., 2002, Thomas et al., 1995). GLUT2 has been shown to be the primary glucose transporter and sensor in rodent pancreatic islets, whereas studies in humans have suggested that GLUT1 plays an important role in facilitating glucose entry into the developing beta cell (De Vos et al., 1995, McCulloch et al., 2011). An example of a difference at the level of the gene structure is the insulin gene: in humans preproinsulin is encoded by a single gene (*INS*), while in mice two functional copies of the gene exist (*Ins1* and *Ins2*).

Studies investigating transcriptional regulation of beta cell development in mice have, however, been successful in identifying pancreatic transcription factor genes important for human pancreatic development. Much of the mouse work has involved the complete or targeted (conditional) knockout of a gene followed by detailed phenotypic studies during embryonic development and, when viable, in the resulting live offspring. To date, mutations in eight different pancreatic transcription factor genes (*PDX1*, *PTF1A*, *GLIS3*, *PAX6*, *RFX6*, *NEUROD1*, *NEUROG3*, *GATA6*) have been identified in patients with neonatal diabetes (Lango Allen et al., 2012, Rubio-Cabezas et al., 2010, Rubio-Cabezas et al., 2011, Sellick et al., 2004, Senée et al., 2006, Smith et al., 2010, Solomon et al., 2009, Stoffers et al., 1997). For some transcription factor genes the genetic etiology in the patients was initially recognized from the similarity to the mouse phenotype (Rubio-Cabezas et al., 2010, Stoffers et al., 1997). Mutations causing a pancreatic/diabetes phenotype have not been found in man for many pancreatic transcription factors important for rodent pancreatic development, but there have been no large, systematic studies of these genes in appropriate patients to date.

The aim of our study was to perform a comprehensive search for recessive mutations in genes encoding transcription factors known to be critical for pancreatic development in mice in a large collection of PNDM patients born to consanguineous parents. Patients with consanguineous parents were chosen, as the likelihood of a recessive cause of their PNDM is greatly increased (Rubio-Cabezas et al., 2009), and all mouse models of transcription factors influencing pancreatic development involve biallelic inactivation. We tested for mutations in homozygous regions encompassing known transcription factor genes independently of the clinical features to avoid the possible bias introduced when clinical features guide candidate gene testing.

## Results and Discussion

We studied 147 patients from consanguineous pedigrees. Mutations in non-transcription factor genes were identified in 110 probands (75%) (Table 1, Figure S1, and Table S1). Systematic investigation of 29 transcription factor genes important for murine pancreatic development identified homozygous mutations in 7 genes in 12 patients from 11 families (30% of the undiagnosed probands or 7.5% of the entire cohort) (Table 1). Overall, 106 out of 121 (88%) patients with a known genetic etiology had homozygous mutations, confirming that neonatal diabetes in the offspring of consanguineous families is usually recessive.

**Table 1:** Pancreatic Transcription Factor Gene Analysis in Patients with Neonatal Diabetes, Related to Tables S1 and S2

Gene	Diabetes Phenotype of the Null Mouse	Number of Homozygous Regions in 37 Patients	Mutations Identified in PNDM Patients	Homozygous Region Coordinates	Homozygous Mutations Identified	Reference(s) in which Patient Was Previously Reported
*FOXA1*	–	2	0	–	–	–
*FOXA2*	embryonic lethal	1	0	–	–	–
*GATA4*	embryonic lethal	2	0	–	–	–
*GATA6*	embryonic lethal	3	0	–	–	–
*GLIS3*	neonatal diabetes	4	2	chr9: 36,587–4,187,290 chr9: 39,094,547–46,744,084	p.? (c.1.−?_388 + ?del) p.G311fs (c.932 delG)	Dimitri et al., 2011
*HES1*	pancreatic hypoplasia	2	0	–	–	–
*HHEX*	pancreatic hypoplasia	1	0	–	–	–
*HNF1B*	embryonic lethal	1	0	–	–	–
*INSM1*	neonatal lethal	2	0	–	–	–
*ISL1*	pancreatic hypoplasia	2	0	–	–	–
*MAFA*	diabetes at 8–12 weeks	0	0	–	–	–
*MAFB*	neonatal lethal	4	0	–	–	–
*MNX1*	pancreatic hypoplasia	10	2	chr7: 147,339,570–158,659,419 chr7: 155,022,291–158,811,981	p.F248L (c.744C > G) p.F272L (c.816C > A)	Bonnefond et al., 2013
*NEUROD1*	neonatal diabetes	3	1	–	p.D122fs (c.364 dupG)	Rubio-Cabezas et al., 2010
*NEUROG3*	neonatal diabetes	1	0	–	–	–
*NKX2.2*	neonatal diabetes	2	2	chr20: 4,435,760–60,974,283 chr20: 19,494,958–32,325,488	p.P119fs (c.356 del) p.R129X (c.385C > T)	–
*NKX3.2*		4	0	–	–	–
*NKX6.1*	reduced insulin secretion	2	0	–	–	–
*NKX6.2*		4	0	–	–	–
*ONECUT1*	adult-onset diabetes	3	0	–	–	–
*PAX4*	neonatal diabetes	1	0	–	–	–
*PAX6*	neonatal lethal	0	0	–	–	–
*PDX1*	pancreatic agenesis	4	2	chr13: 27,258,487–42,966,967 chr13: 21,702,312–43,640426	p.A152G (c.455C > G) p.R176Q (c.527G > A)	De Franco et al., 2013
*PTF1A*	pancreatic agenesis	8	1	chr10: 14,877,485–49,578,351	p.? (c.784 + 4A > G)	Lango Allen et al., 2012
*RBPJ*	embryonic lethal	2	0	–	–	–
*RBPJL*		4	0	–	–	–
*RFX6*	neonatal diabetes	5	1	chr6: 109,613,154–144,987,523	p.S217P (c.649T > C)	Smith et al., 2010
*SOX17*	embryonic lethal	1	0	–	–	–
*SOX9*	embryonic lethal	6	0	–	–	–

**Total: 29**	–	**Total: 84**	**Total: 11**	–	–	–

Transcription factor genes important for murine pancreatic development and the resulting diabetes phenotype in null mice. Genome-wide SNP typing identified 84 homozygous regions encompassing a transcription factor gene in 30 patients with permanent neonatal diabetes. Using Sanger sequencing, 11 mutations were detected in 7 of these genes.

### Homozygous Mutations in *NKX2-2* Cause Neonatal Diabetes

**Figure 1:**
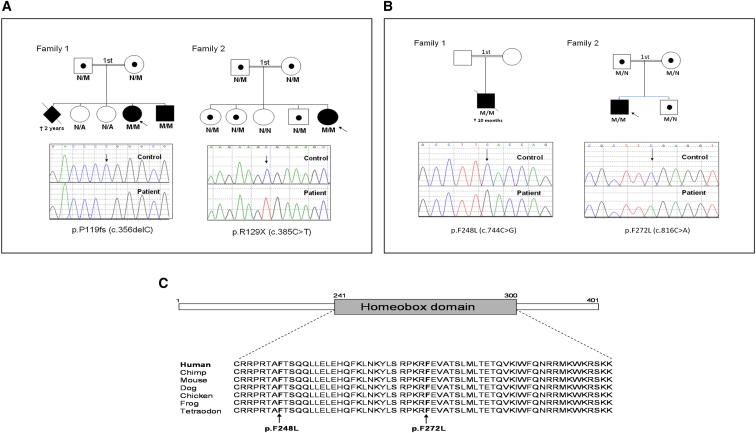
*NKX2-2* and *MNX1* Mutations in Four Families with Neonatal Diabetes (A) Partial pedigrees of two families in which *NKX2-2* mutations were identified. Below each pedigree is an electropherogram depicting the homozygous mutation identified in each proband. N/A, not available. (B) Partial pedigrees of two families with *MNX1* mutations. Below each pedigree is an electropherogram depicting the homozygous mutation identified in each proband. (C) The highly conserved sequence of the homeobox domain within MNX1 is provided for various species. An arrow points to the residues found to be mutated in the two probands with permanent neonatal diabetes. See also Figure S1.

Homozygous *NKX2-2* nonsense or frameshift mutations were identified in three patients from two families (Table 2). These mutations are highly likely to be pathogenic, as they are null mutations. Cosegregation studies were consistent with recessive inheritance (Figure 1A). No heterozygous or homozygous truncating mutations have been reported in 6,500 individuals sequenced by the NHLBI Exome Sequencing Project (http://evs.gs.washington.edu/EVS/). All three patients have severe defects in insulin secretion, as shown by intrauterine growth retardation (IUGR) and presentation of diabetes at an early age without features of pancreatic exocrine dysfunction (Table 2). This is very similar to mice that are homozygous for a targeted disruption of *Nkx2-2* and die shortly after birth with severe hyperglycemia (Briscoe et al., 1999, Sussel et al., 1998) (Table 2). They have normal exocrine function, lack beta cells, and have fewer alpha and pancreatic polypeptide cells (Sussel et al., 1998). The patients with homozygous null mutations in *NKX2-2* also have severe developmental delay, which affects both motor and intellectual function as well as more specific features, such as hypotonia, cortical blindness, impaired visual tracking, and hearing impairment. These are consistent with the severe neurological features seen in the *Nkx2-2* knockout mouse (Table 2) and *Nkx2-2* being essential for hindbrain development, ventral neuronal patterning, and oligodendrocyte differentiation (Briscoe et al., 1999, Qi et al., 2001).

**Table 2:** Clinical Characteristics of Patients with Homozygous *NKX2-2* or *MNX1* Mutations and Comparisons with Mouse Model, Related to Table S3

NKX2-2	Homozygous Null Mouse∗	Humans		
		Proband 1 (p.P119fs)	Sibling (p.P119fs)	Proband 2 (p.R129X)
Gender	–	female	male	female
Current age (years)	–	5.5	1.5	13
Birth weight (gestation)	–	1.36 kg (35 weeks)	1.68 kg (40 weeks)	1.22 kg (37 weeks)
Birth weight (SDS)	not distinguishable from wild-type littermates at birth	−2.8	−3.64	−4.52

**Pancreatic Features**				

Age diagnosis (days)	2	2	2	7
Glucose at presentation (mmol/l)	23 (4.5 wild-type littermates)	18	not available	67
Current insulin requirement (U/kg/day)	–	0. 57	0.60	1.2
Current HbA1c (%) (mmol/mol)	–	9% (75)	7.9% (63)	9.5%–12% (80–108)
Evidence of exocrine insufficiency	no	no	no	no

**Extrapancreatic Features**				

CNS	retarded oligodendrocyte differentiation and absence of hindbrain serotonergic neurons	severe developmental delay (unable to stand or talk at 5 years of age), hypotonia, cortical blindness, thin corpus callosum and generalized gliosis on MRI	severe developmental delay (motor function is that of a 5 month old at 9 months of age)	moderate developmental delay (walks with assistance at 11 years of age), hypotonia, inability to fix gaze and follow, bilateral hearing impairment
Growth	growth retarded	short stature (−3 SDS)	–	short stature (−2.2 SDS)
Additional features	none reported	swallowing difficulties, severe constipation	constipation	none reported

MNX1	Homozygous Null Mouse∗	Humans	
		Proband 1 (p.F248L)	Proband 2 (p.F272L)
Gender	–	male	female
Current Age	–	deceased at an age of 10 months	3 years
Birth weight (gestation)	IUGR	2.23 kg (40 weeks)	1.90 kg (38 weeks)
Birth weight (SDS)	–	−2.54	−3.09

**Pancreatic Features**			

Age at diagnosis of diabetes (weeks)	dorsal lobe agenesis, small islets, reduced number of beta cells	30	1
Glucose at presentation (mmol/l)	–	12.1	35
Insulin requirement	–	0.3 U/kg/day	0.8 U/kg/day
HbA1c % (mmol/mol)	–	6.6% (49)	7.8% (62)
Evidence of exocrine insufficiency	no	no	no

**Extrapancreatic Features**			

CNS	aberrant motor neuron differentiation, absent phrenic nerve	severe developmental delay, poor suck and swallow, neurogenic bladder, flexion deformities of legs, MRI scan of brain demonstrated generalized reduction in myelin	none reported
Muscular/skeletal	–	short stature (<3^rd^ percentile)	normal stature (59^th^ percentile)
Lung	pulmonary paralysis and lung atelectasis (neonatal lethal)	hypoplastic lungs requiring CPAP	none reported
Additional features	not reported	rocker bottom feet, poorly developed renal cortex and medulla, sacral agenesis, high imperforate anus	failure to thrive (current weight 11.7kg, <5^th^ percentile)

∗ Mouse data reported in Arber et al., 1999, Briscoe et al., 1999, Harrison et al., 1999, Qi et al., 2001, Sussel et al., 1998, and Thaler et al. (1999). SDS, SD scores; IUGR, intrauterine growth retardation; CPAP, continuous positive airway pressure.

### Homozygous Mutations in *MNX1* Cause Neonatal Diabetes

Two probands have homozygous missense mutations in *MNX1* (previously described as *HLXB9*). The p.F248L and p.F272L missense mutations are highly likely to be pathogenic, as they cosegregate with neonatal diabetes (Figure 1B), affect highly conserved residues within the homeodomain (Table 2, Figure 1C), are not listed in 6,500 individuals sequenced by the NHLBI Exome Sequencing Project, and in silico analysis predicted the mutations to be disease causing. Our results are in line with the recently published paper by Bonnefond et al. (2013), who independently found the same *MNX1* p.F272L mutation in proband 2. Both probands in our study had severe intrauterine growth retardation and were diagnosed with diabetes in infancy without evidence of exocrine pancreatic dysfunction. This is similar to the *Mnx1* null mice that are born smaller than their wild-type littermates and have small islets and reduced numbers of beta cells and normal exocrine function (Table 2) (Harrison et al., 1999). These mice also have dorsal lobe agenesis of the pancreas; it is not known if this is a feature of our two patients, as imaging and post mortem investigations were not performed. Proband 1 (but not proband 2) had marked extrapancreatic developmental features, including severe neurological complications and hypoplastic lungs (respiratory failure caused death at 10 months) (Table 2). In keeping with these observations, *Mnx1* null mice have aberrant motor neuron differentiation but are difficult to study as they die at birth due to respiratory paralysis, which probably results from failure in the development of the phrenic nerve (Thaler et al., 1999). The variability in phenotype between the two probands could be due to differences in the severity of their mutations, but further studies investigating the functional consequences of these missense mutations are required in order to explore this hypothesis.

Proband 1 also had sacral agenesis and a high imperforate anus, which are both cardinal features of Currarino syndrome (OMIM #176450), resulting from heterozygous *MNX1* mutations (Currarino et al., 1981, Ross et al., 1998). Mice homozygous for a null mutation do not have skeletal defects (Harrison et al., 1999). Proband 2 and his heterozygous relatives do not have features of Currarino syndrome. The highly variable phenotype is not unexpected, as around 50% of heterozygous carriers are clinically asymptomatic (Köchling et al., 2001). As diabetes is not a feature of Currarino syndrome, it seems likely that a single copy of *MNX1* is sufficient to maintain normal pancreatic development and glucose homeostasis, a hypothesis that is further supported by the observation that heterozygous mice have a normal pancreas (Harrison et al., 1999).

### Known Causes of Neonatal Diabetes

Homozygous mutations in five pancreatic transcription factor genes previously reported to cause PNDM were identified in seven probands. The phenotype of these patients is similar to that seen in mice with biallelic inactivation of these genes (Table 3). Our two patients with *GLIS3* mutations, the six previous cases (Dimitri et al., 2011, Senée et al., 2006), and the *Glis3* mouse knockout (Watanabe et al., 2009) all have neonatal diabetes diagnosed in the first week of life and congenital hypothyroidism. We identified one patient with a homozygous *NEUROD1* mutation. Both this patient and a previously reported case from a nonconsanguineous pedigree (Rubio-Cabezas et al., 2010) have neonatal diabetes, neurodevelopmental delay, cerebellar hypoplasia, and hearing and visual impairment. The *Neurod1* null mouse also has neonatal diabetes and severe neurological defects, including impaired coordination and ataxia, cerebellar hypoplasia, sensorineural deafness, and retinal blindness (Liu et al., 2000, Miyata et al., 1999, Morrow et al., 1999, Naya et al., 1997, Pennesi et al., 2003). The proband with a homozygous *RFX6* mutation was diagnosed with diabetes at 8 days and had duodenal atresia and gall bladder agenesis. This is consistent with the previous five cases and the *Rfx6* knockout mouse, which has neonatal diabetes and small bowel atresia (Smith et al., 2010).

**Table 3:** A Comparison of the Phenotype Observed in the Patients with Homozygous Mutations in Known Pancreatic Transcription Factors to the Phenotype Observed in Homozygous Inactivation in Mice

Gene	Mutation	Patient		Mouse		References
		Pancreas	Additional Features	Pancreas	Additional Features	
*GLIS3*	c.1.−?_388 + ?del^1^	neonatal diabetes	renal cystic dysplasia, congenital hypothyroidism, hepatic fibrosis	null: neonatal diabetes	null: neonatal lethality, polycystic kidney disease, congenital hypothyroidism	Watanabe et al., 2009
	c.932 delG	neonatal diabetes	multiple small renal cysts, congenital hypothyroidism, osteopenia, anemia			
*NEUROD1*	c.364 dupG^2^	neonatal diabetes	learning difficulties, severe cerebellar hypoplasia, sensorineural deafness, retinal dystrophy	null: neonatal diabetes	null: cerebellar hypoplasia, impaired coordination and ataxia, impaired hearing and balance, retinal degeneration, blindness, and seizures	Kim et al., 2001, Liu et al., 2000, Morrow et al., 1999, Naya et al., 1997
*PDX1*	p.A152G^3^	neonatal diabetes	none reported	null: pancreatic agenesis hypomorphic mutation: neonatal diabetes	null: abnormal duodenum and stomach morphology hypomorphic mutation: none reported	Jonsson et al., 1994, Offield et al., 1996, Oliver-Krasinski et al., 2009, Stoffers et al., 1997
	p.R176Q^3^	neonatal diabetes	none reported			
*PTF1A*	c.784 + 4A > G^4^	pancreatic agenesis	none reported	null: pancreatic agenesis hypomorphic mutation: pancreatic hypoplasia	null: cerebellar hypoplasia hypomorphic mutation: cerebellar hypoplasia	Fukuda et al., 2008, Hoshino et al., 2005, Kawaguchi et al., 2002, Krapp et al., 1998, Sellick et al., 2004
*RFX6*	p.S217P^5^	neonatal diabetes	duodenal atresia, gall bladder agenesis	null: neonatal diabetes	null: small bowel atresia	Smith et al., 2010

Some patients have been reported previously by Dimitri et al., 2011, Rubio-Cabezas et al., 2010, De Franco et al., 2013, Lango Allen et al., 2012, and Smith et al. (2010).

Two probands with homozygous *PDX1* (previously described as *IPF1*) missense mutations had isolated PNDM. In both man and mouse, the severity of the *PDX1* mutation determines the pancreatic phenotype. Pancreatic agenesis was seen in the mouse knockout (Jonsson et al., 1994) and in patients with two copies of a severe mutation (Fajans et al., 2010, Schwitzgebel et al., 2003, Stoffers et al., 1997, Stoffers et al., 1998, Thomas et al., 2009). In mice, hypomorphic mutations are associated with diabetes without evidence of exocrine deficiency (Oliver-Krasinski et al., 2009); we and others have shown that patients with less-severe homozygous missense mutations have neonatal diabetes with normal or mildly impaired exocrine function (De Franco et al., 2013, Nicolino et al., 2010).

An intronic *PTF1A* mutation (c.784 + 4A > G) was identified in a proband with pancreatic agenesis. This mutation is predicted to result in an alternative splice donor site (Alamut Interactive Biosoftware, version 2.1) and the inclusion of an additional 4 bases in the mRNA, causing a frameshift and premature termination codon at residue 271. The pancreatic phenotype is similar to the three previously reported patients with nonsense or frameshift mutations and the *Ptf1a* null mouse (Al-Shammari et al., 2011, Krapp et al., 1998, Sellick et al., 2004). Our patient has mild learning difficulties at 15 years old, which is in contrast to the other patients who had severe neurological features, causing death before 4 months of age. These patients and the knockout mouse had cerebellar aplasia. In a mouse model, reduced *Ptf1a* gene dosage results in pancreatic hypoplasia and glucose intolerance in a dose-dependent manner (Fukuda et al., 2008). We hypothesize that the absence of cerebellar agenesis in this patient may reflect a reduced functional severity of the splicing mutation compared to previously reported null mutations.

The finding that mutations in *NKX2-2* and *MNX1* can cause neonatal diabetes takes the number of genes in which mutations are known to cause PNDM from 18 to 20, of which 10 encode pancreatic transcription factors. Unlike previous studies, which have identified mutations in humans because of similarities in phenotype with the null mouse, our systematic approach guided by areas of linkage has allowed us to screen all transcription factor genes independently of clinical features. Our results confirm that the consequence of inactivation of pancreatic transcription factor genes in humans is similar to the phenotype observed in knockout mice. This is in contrast to heterozygous loss-of-function mutations where differences in phenotype have been observed. For example, monoallelic mutations in *GATA6* or *HNF1B* cause pancreatic agenesis with congenital heart defects or renal cysts with diabetes, respectively, while mice heterozygous for a null mutation in *Gata6* or *Hnf1b* show no obvious phenotype (Bingham et al., 2001, Haumaitre et al., 2005, Lango Allen et al., 2012, Morrisey et al., 1998).

There are some limitations to our study. The minimum prevalence of transcription factor mutations in our cohort of patients with consanguineous PNDM was 7.5%, but the true prevalence may be slightly higher since heterozygous or compound heterozygous mutations would have been missed as a result of the study design. However, as all of the patients were known to be consanguineous, the prior probability is that they have recessively inherited disease due to a homozygous mutation. While the identification of patients with homozygous inactivating mutations in pancreatic transcription factor genes has confirmed their role in human organogenesis, these findings can only provide limited information on pancreatic development, as detailed physiological studies in man are not always possible. This is especially true for the patients within our cohort with *NKX2*-2 and *MNX1* mutations that are all currently under 13 years of age and, for those with *NKX2*-2 mutations, have severe developmental delay, which means that in-depth studies on pancreatic and neurological function are not possible.

In conclusion, we have shown that mutations in *NKX2*-2 and *MNX1* cause neonatal diabetes. This confirms a key role for NKX2-2 and MNX1 in human pancreatic development. Comparisons of phenotypes observed in humans and mice with inactivation of transcription factor genes supports common steps critical for pancreatic development. This study validates the use of knockout mice for understanding beta cell development in humans.

## Experimental Procedures

### Cohort

We recruited 147 patients from consanguineous pedigrees (parents of the proband were second cousins or more closely related) who were diagnosed with permanent diabetes before 6 months of age or were diagnosed with permanent diabetes before 9 months and had any additional clinical features that made a diagnosis of type 1 diabetes less likely. Informed consent was obtained from all participants or their parents, and institutional review board approval was received for this study.

### Exclusion of Nontranscription Factor Mutations in PNDM

To detect mutations in non-transcription factor genes that cause PNDM, we used Sanger sequencing (*ABCC8*, *KCNJ11*, *INS*) in all patients, followed by homozygosity mapping with sequencing of known genes (*EIF2AK3*, *IER3IP1*, *GCK*, *SLC19A2*, and *SLC2A2*) within homozygous regions >3 Mb.

### Detecting Mutations of Pancreatic Transcription Factor Genes

From published literature, we identified 29 transcription factor genes crucial for murine pancreatic development (Seymour and Sander, 2011, Zaret and Grompe, 2008) (Table 1). We identified homozygous regions through genome-wide single nucleotide polymorphism (SNP) genotyping and used Sanger sequencing to detect mutations within homozygous regions encompassing these genes.

### Genome-wide SNP Analysis to Localize Etiological Genes by Linkage

Genotyping was undertaken on the Affymetrix 10K, 500K, or v6.0 SNP chips by Medical Solutions, ALMAC Diagnostics, or AROS Applied Biotechnology. Processing of genomic DNA was performed as per the Affymetrix protocol, and in-house Perl scripts were developed to identify regions of significant homozygosity (>3 Mb) as previously described (Flanagan et al., 2011).

### Sequencing of Pancreatic Transcription Factor Genes

When a large homozygous region encompassing one of the pancreatic transcription factor genes was identified, the coding regions and intron and exon boundaries of the gene were sequenced (Table 1, Figure S1, and Tables S2 and S3). Details of PCR primers and sequencing conditions are available on request. Pathogenicity was assessed on the basis of the characteristics of the mutation, the conservation of the affected amino acid, the absence from variant databases (accessed using Alamut v.2 Interactive Biosoftware), and cosegregation within families.

### Patient Phenotype Assessment

For patients with homozygous mutations, their phenotype (both pancreatic and extrapancreatic) was compared with the phenotype of the knockout mouse for the same gene.
