# Supplementary material for: Analysis of Transcription Factors Key for Mouse Pancreatic Development Establishes NKX2-2 and MNX1 Mutations as Causes of Neonatal Diabetes in Man
Source: Cell Metab. 2014 Jan 7;19(1):146–54. doi: 10.1016/j.cmet.2013.11.021 (PMC3887257; doi:10.1016/j.cmet.2013.11.021)
Supplement: Document S1. Figure S1 and Tables S1–S3 [file mmc1.pdf]

Cell Metabolism, Volume *19*

Supplemental Information

Analysis of Transcription Factors Key for Mouse Pancreatic Development Establishes *NKX2-2* and *MNX1* Mutations as Causes of Neonatal Diabetes in Man

Sarah E. Flanagan, Elisa De Franco, Hana Lango Allen, Michele Zerah, Majedah M. Abdul-Rasoul, Julie A.Edge, Helen Stewart, Elham Alamiri, Khalid Hussain, Sam Wallis, Liat de Vries, Oscar Rubio-Cabezas, Jayne A.L. Houghton, Emma L. Edghill, Ann-Marie Patch, Sian Ellard, and Andrew T. Hattersley

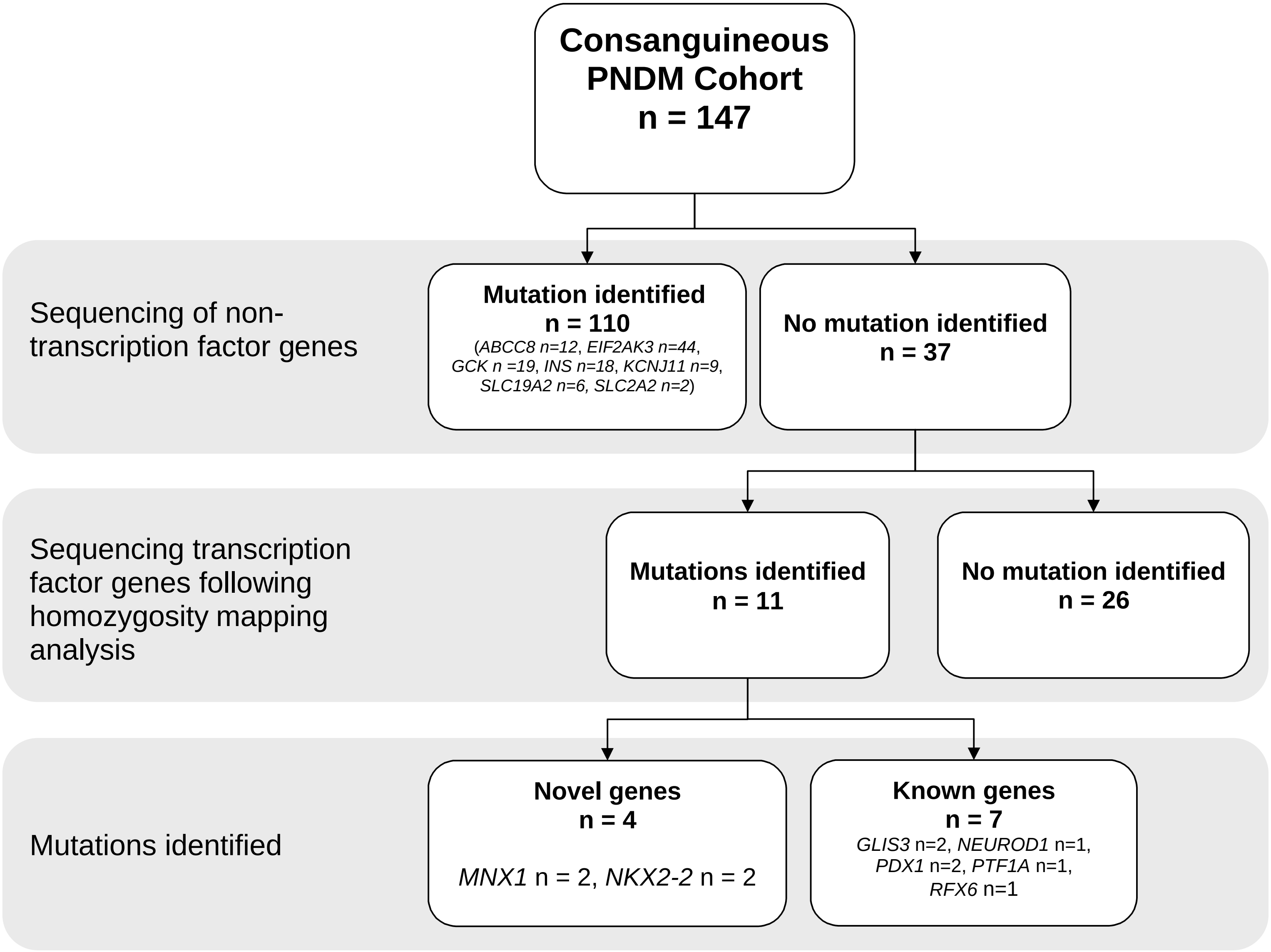

Figure S1 (related to figure 1): Schematic representation of the order and results of genetic testing in 147 consanguineous probands with permanent neonatal diabetes (PNDM)

	Gene	Mutation Details			Patient previously reported
		Protein Change	Nucleotide change	Zygosity	
1	*ABCC8*	p.D209E	c.745G>A	Heterozygous	Novel
2	*ABCC8*	p.P254S	c.760C>T	Homozygous	Novel
3	*ABCC8*	p.P254S	c.760C>T	Homozygous	Novel
4	*ABCC8*	p.E382K	c.1144G>A	Homozygous	(Ellard et al., 2007)
5	*ABCC8*	p.E382V	c.1145A>T	Homozygous	Novel
6	*ABCC8*	p.W688R	c.2062T>C	Homozygous	Novel
7	*ABCC8*	p.W688R	c.2062T>C	Homozygous	Novel
8	*ABCC8*	p.R826W	c.2476C>T	Heterozygous	Novel
9	*ABCC8*	p.R826W	c. 2476C>T	Homozygous	Novel
10	*ABCC8*	p.F1164L	c.3490T>C	Homozygous	Novel
11	*ABCC8*	p.A1264E	c.3791C>T	Homozygous	Novel
12	*ABCC8*	p.A1264E	c.3791C>T	Homozygous	Novel
13	*EIF2AK3*	p.K150RfsX2	c.449del	Homozygous	(Rubio-Cabezas et al., 2009)
14	*EIF2AK3*	p.Q165X	c.493C>T	Homozygous	Novel
15	*EIF2AK3*	p.Y213C	c.638A>G	Homozygous	Novel
16	*EIF2AK3*	p.S312X	c.935C>A	Homozygous	(Rubio-Cabezas et al., 2009)
17	*EIF2AK3*	p.Q333X	c.997C>T	Homozygous	Novel
18	*EIF2AK3*	p.K345X	c.1032dupT	Homozygous	(Rubio-Cabezas et al., 2009)
19	*EIF2AK3*	p.V349SfsX3	c.1044_1057del	Homozygous	(Rubio-Cabezas et al., 2009)
20	*EIF2AK3*	p.N420TfsX14	c.1259delA	Homozygous	(Rubio-Cabezas et al., 2009)
21	*EIF2AK3*	p.N420TfsX14	c.1259delA	Homozygous	(Al-Shawi et al., 2012)
22	*EIF2AK3*	p.L425X	c.1274T>A	Homozygous	Novel
23	*EIF2AK3*	p.L425X	c.1274T>A	Homozygous	(Rubio-Cabezas et al., 2009)
24	*EIF2AK3*	p.W430X	c.1290G>A	Homozygous	(Rubio-Cabezas et al., 2009)
25	*EIF2AK3*	p.W430X	c.1290G>A	Homozygous	Novel
26	*EIF2AK3*	p.S469X	c.1406C>G	Homozygous	(Rubio-Cabezas et al., 2009)
27	*EIF2AK3*	p.R491X	c.1471C>T	Homozygous	(Mihci et al., 2012)
28	*EIF2AK3*	p.W520X	c.1560G>A	Homozygous	Novel
29	*EIF2AK3*	p.W521X	c.1562G>A	Homozygous	(Rubio-Cabezas et al., 2009)
30	*EIF2AK3*	p.W521X	c.1562G>A	Homozygous	Novel
31	*EIF2AK3*	p.E523X	c.1567_1570del	Homozygous	(Rubio-Cabezas et al., 2009)
32	*EIF2AK3*	p.R587X	c.1759C>T	Homozygous	Novel
33	*EIF2AK3*	p.R587L	c.1760G>T	Homozygous	Novel
34	*EIF2AK3*	p.Y588X	c.1764T>G	Homozygous	Novel
35	*EIF2AK3*	p.R632W	c.1894C>T	Homozygous	Novel

36	*EIF2AK3*	p.R637X	c.1909C>T	Homozygous	Novel
37	*EIF2AK3*	p.V639I	c.1915G>A	Homozygous	Novel
38	*EIF2AK3*	p.I650T	c.1949T>C	Homozygous	(Rubio-Cabezas et al., 2009)
39	*EIF2AK3*	p.S718TfsX6	c.2153del	Homozygous	(Rubio-Cabezas et al., 2009)
40	*EIF2AK3*	p.C768X	c.2304_2305del	Homozygous	(Rubio-Cabezas et al., 2009)
41	*EIF2AK3*	p.R902X	c.2704C>T	Homozygous	Novel
42	*EIF2AK3*	p.R902X	c.2704C>T	Homozygous	Novel
43	*EIF2AK3*	p.G956E	c.2867G>A	Homozygous	(Rubio-Cabezas et al., 2009)
44	*EIF2AK3*	p.G956E	c.2867G>A	Homozygous	Novel
45	*EIF2AK3*	p.G985R	c.2953G>A	Homozygous	(Rubio-Cabezas et al., 2009)
46	*EIF2AK3*	p.Y989C	c.2966A>G	Homozygous	Novel
47	*EIF2AK3*	p.S991N	c.2972G>A	Homozygous	Novel
48	*EIF2AK3*	p.?	c.2981+1dupG	Homozygous	(Rubio-Cabezas et al., 2009)
49	*EIF2AK3*	p.?	c.2981+1G>A	Homozygous	Novel
50	*EIF2AK3*	p.G1010D	c.3029G>A	Homozygous	Novel
51	*EIF2AK3*	p.L1030X	c.3087delC	Homozygous	Novel
52	*EIF2AK3*	p.D1032LfsX16	c.3094_3098del	Homozygous	Novel
53	*EIF2AK3*	p.R1064X	c.3190C>T	Homozygous	Novel
54	*EIF2AK3*	p.R1064X	c.3190C>T	Homozygous	(Rubio-Cabezas et al., 2009)
55	*EIF2AK3*	p.R1064X	c.3190C>T	Homozygous	Novel
56	*EIF2AK3*	p.R1064X	c.3190C>T	Homozygous	Novel
57	*GCK*	p.E40K	c.118G>A	Homozygous	Novel
58	*GCK*	p.H50D	c.148C>G	Homozygous	Novel
59	*GCK*	p.G72R	c.214G>A	Homozygous	Novel
60	*GCK*	p.L146P	c.437T>C	Homozygous	Novel
61	*GCK*	p.S151T	c.451T>A	Homozygous	Novel
62	*GCK*	p.T168A	c.502A>G	Homozygous	(Turkkahraman et al., 2008)
63	*GCK*	p.K169R	c.506A>G	Homozygous	Novel
64	*GCK*	p.A208T	c.622G>A	Homozygous	Novel
65	*GCK*	p.C220R	c.658T>C	Homozygous	Novel
66	*GCK*	p.G261R	c.781G>A	Homozygous	Novel
67	*GCK*	p.M393T	c.1178T>C	Homozygous	Novel
68	*GCK*	p.R397L	c.1190G>T	Homozygous	Novel
69	*GCK*	p.R397L	c.1190G>T	Homozygous	Novel
70	*GCK*	p.R397L	c.1190G>T	Homozygous	(Porter et al., 2005)
71	*GCK*	p.F419SfsX12	c.1256delT	Homozygous	Novel
72	*GCK*	p.S441L	c.1322C>T	Homozygous	Novel
73	*GCK*	p.A449T	c.1345G>A	Homozygous	Novel
74	*GCK*	p.A449T	c.1345G>A	Homozygous	Novel
75	*GCK*	p.A449T	c.1345G>A	Homozygous	Novel

76	*INS*	p.?	c.-370-?_186+?del	Homozygous	(Garin et al., 2010)
77	*INS*	p.?	c.-331C>G	Homozygous	(Garin et al., 2010)
78	*INS*	p.?	c.-331C>G	Homozygous	Novel
79	*INS*	p.?	c.-331C>G	Homozygous	Novel
80	*INS*	p.?	c.-331C>G	Homozygous	Novel
81	*INS*	p.?	c.-331C>G	Homozygous	(Garin et al., 2010)
82	*INS*	p.?	c.-331C>G	Homozygous	(Garin et al., 2010)
83	*INS*	p.?	c.-331C>A	Homozygous	Novel
84	*INS*	p.0?	c.1-?_330+?del	Homozygous	Novel
85	*INS*	p.0?	c.1-?_330+?del	Homozygous	Novel
86	*INS*	p.0?	c.3G>A	Homozygous	(Garin et al., 2010)
87	*INS*	p.0?	c.3G>T	Homozygous	(Garin et al., 2010)
88	*INS*	p.L39P	c.116 T>C	Heterozygous	Novel
89	*INS*	p.F48C	c.143T>G	Heterozygous	Novel
90	*INS*	p.Q62X	c.184C>T	Homozygous	(Garin et al., 2010)
91	*INS*	p.R89C	c.265C>T	Heterozygous	Novel
92	*INS*	p.L105P	c.314T>C	Heterozygous	Novel
93	*INS*	p.C109Y	c.326G>A	Heterozygous	Novel
94	*KCNJ11*	p.R50Q	c.149G>A	Heterozygous	Novel
95	*KCNJ11*	p.V59M	c.175G>A	Heterozygous	Novel
96	*KCNJ11*	p.R201C	c.601C>T	Heterozygous	Novel
97	*KCNJ11*	p.R201C	c.601C>T	Heterozygous	Novel
98	*KCNJ11*	p.R201C	c.601C>T	Heterozygous	Novel
99	*KCNJ11*	p.R201H	c.601C>T	Heterozygous	Novel
100	*KCNJ11*	p.R201H	c.602G>A	Heterozygous	Novel
101	*KCNJ11*	p.R201H	c.602G>A	Heterozygous	Novel
102	*KCNJ11*	p.G334V	c.1001G>T	Heterozygous	Novel
103	*SLC19A2*	p.Y79X	c.237C>A	Homozygous	(Shaw-Smith et al., 2012)
104	*SLC19A2*	p.G105E	c.314G>A	Homozygous	Novel
105	*SLC19A2*	p.I109MXfs27	c.327_334del	Homozygous	(Shaw-Smith et al., 2012)
106	*SLC19A2*	p.S143F	c.428C>T	Homozygous	(Shaw-Smith et al., 2012)
107	*SLC19A2*	p.S214TfsX14	c.641del	Homozygous	Novel
108	*SLC19A2*	p.W320G	c.958T>G	Homozygous	Novel
109	*SLC2A2*	p.F114LfsX16	c.339delC	Homozygous	(Habeb et al., 2012)
110	*SLC2A2*	p.W420L	c.1259G>T	Homozygous	Novel

Table S1 related to table 1: Mutations identified in non-transcription factor genes.

Gene	Genomic Position*	Sequence accession
FOXA1	Chr14:38,066,489-38,057,189	NM_004496
FOXA2	Chr20:22,568,101-22,559,642	NM_021784
GATA4	Chr8:11,559,717-11,619,509	NM_002052
GATA6	Chr18:19,747,416-19,784,227	NM_005257
GLIS3	Chr9:4,302,035-3,822,128	NM_001042413
HES1	Chr3:193,851,931-193,858,401	NM_005524
HHEX	Chr10:94,447,681-94,457,408	NM_002729
HNF1B	Chr17:36,107,096-36,044,434	NM_000458
INSM1	Chr20:20,346,765-20,353,593	NM_002196
ISL1	Chr5:50,676,958-50,692,564	NM_002202
MAFA	Chr8:144,514,602-144,508,230	NM_201589
MAFB	Chr20:39,319,876-39,312,515	NM_005461
MNX1	Chr7:156,805,347-156,795,547	NM_005515
NEUROD1	Chr7:182,547,392-182,538,833	NM_002500
NEURGO3	Chr10:71,335,210-71,329,791	NM_020999
NKX2.2	Chr20:21,496,664-21,489,655	NM_002509
NKX3.2	Chr4:13,548,114-13,540,454	NM_001189
NKX6.1	Chr4:85,421,387-85,412,436	NM_006168
NKX6.2	Chr10:134,601,537-134,596,320	NM_177400
ONECUT1	Chr15:53,084,209-53,047,353	NM_004498
PAX4	Chr7:127,257,780-127,248,346	NM_006193
PAX6	Chr11:31,841,509-31,804,340	NM_000280
PDX1	Chr13:28,492,168-28,502,451	NM_000209
PTF1A	Chr10:23,479,460-23,485,181	NM_178161
RBPJ	Chr4:26,319,332-26,435,278	NM_005349
RBPJL	Chr20:43,933,491-43,498,464	NM_014276
RFX6	Chr6:117,196,376-117,255,326	NM_173560
SOX17	Chr8:55,368,495-55,375,456	NM_022454
SOX9	Chr17:70,115,161-70,124,561	NM_000346

*Hg19 coordinates

Table S2 related to table 1: Hg19 genomic co-ordinates and sequence accession numbers for the 29 pancreatic transcription factor genes analysed for mutations.

Gene	Pancreas	Central Nervous System	Additional Features	References
FOXA1	**KO:** Hypoglycaemia caused by impaired glucagon secretion		**KO:** Abnormal feeding and hypotriglyceridemia Postnatal lethality between days 2-14	(Kaestner et al., 1999; Shih et al., 1999)
FOXA2	**KO:** Embryonic lethal<br>**Conditional KO:** Hypoglycemia	**KO:** Somite & neural tube defects. Absence of floor plate, notochord & motor neurons		(Ang and Rossant, 1994; Sund et al., 2001; Weinstein et al., 1994)
GATA4	**KO:** Embryonic lethal<br>**Conditional KO:** Agenesis of ventral pancreas		**KO:** Defects in ventral morphogenesis, absence of primitive heart tube & foregut. Embryonic lethal<br>**Conditional KO:** Defective liver formation	(Molkentin et al., 1997; Watt et al., 2007)
GATA6	**KO:** Embryonic lethal<br>**Conditional KO:** Pancreatic agenesis		**Conditional KO:** Defective endoderm differentiation and heart tube formation	(Decker et al., 2006; Morrisey et al., 1998; Xin et al., 2006)
HES1	**KO:** Pancreatic hypoplasia	**Conditional KO:** Premature neurogenesis, severe neural tube defects & supernumerary inner ear hair cells	**KO:** Gallbladder agenesis & extrahepatic bile duct hypoplasia	(Ishibashi et al., 1995; Ito et al., 2000; Jensen et al., 2000; Sumazaki et al., 2004; Zine et al., 2001)
HHEX	**KO:** Failure in ventral pancreatic specification	**KO:** Abnormal forebrain development	**KO:** Abnormal vasculogenesis & cardiac development, liver and thyroid dysplasia. Embryonic lethal	(Hallaq et al., 2004; Martinez Barbera et al., 2000)
HNF1B	**KO:** Embryonic lethal<br>**Conditional KO:** Pancreatic agenesis/hypoplasia		**KO:** Impaired embryonic growth & abnormal development of extraembryonic membranes<br>**Conditional KO:** Biliary system and liver dysfunction	(Coffinier et al., 2002; Coffinier et al., 1999; Haumaitre et al., 2005)
INSM1	**KO:** Impaired endocrine cell maturation (~90% reduction on insulin +ve cells)	**KO:** Reduced number of basal progenitors in the neocortex & marked reduction in cortical plate radial thickness	**KO:** Respiratory failure and impaired intestinal endocrine cell development. Perinatal and neonatal lethality	(Gierl et al., 2006; Rosenbaum et al., 2011)
ISL1	**KO:** Pancreatic hypoplasia	**KO:** Absence of motor neurons	**KO** Heart malformations, lethal by embryonic day 11.5	(Ahlgren et al., 1997; Pfaff et al., 1996)
MAFA	**KO:** Glucose intolerance, diabetes at 8-12 weeks			(Zhang et al., 2005)
MAFB	**KO:** Reduced numbers of alpha and beta cells	**KO:** Segmentation defects in the caudal hindbrain, loss of facial motor neurons, impaired inner ear development	**KO:** Arrested maturation of kidney podocytes and central apnes. Neonatal lethal	(Artner et al., 2007; Blanchi et al., 2003; Choo et al., 2006; Moriguchi et al., 2006)
NKX3.2			**KO:** Skeletal dysplasia, absent spleen & gastroduodenal tract malformation. Perinatal lethal	(Akazawa et al., 2000)
NKX6.1	**KO:** Strongly reduced beta cell number and insulin secretion	**KO:** Abnormal neuron specification, decreased motor neuron number	**KO:** Neonatal lethality	(Sander et al., 2000)
NKX6.2		**KO:** Impaired coordination, axon degeneration in the optic nerve		(Southwood et al., 2004)
ONECUT1	**KO:** Adult-onset diabetes		**KO:** Growth retardation, biliary tract defects, absent gallbladder	(Clotman et al., 2002; Jacquemin et al., 2000)
PAX4	**KO:** Neonatal diabetes. Absence of mature beta cells	**KO:** Postnatal lethal		(Sosa-Pineda et al., 1997)
RBPJ	**KO:** Embryonic lethal<br>**Conditional KO:** Pancreatic hypoplasia. Postnatal lethal because of exocrine insufficiency	**KO:** Microencephaly	**KO:** Defective cranial-bone formation. Embryonic lethal.<br>**Conditional KO:** Arteriovenous malformations, hepidermis/hair defects	(Mead and Yutzey, 2012; Nakhai et al., 2008; Oka et al., 1995)
RBPJL	**KO:** Reduced size of acinar cells			(Masui et al., 2010)
SOX17	**KO:** Embryonic lethal<br>**Conditional KO:** Ectopic pancreatic tissue in liver		**Conditional KO:** Liver hypoplasia, gallbladder agenesis, severe hematopoietic defects	(Kim et al., 2007; Spence et al., 2009)
SOX9	**Conditional KO:** pancreatic hypoplasia			(Bi et al., 2001; Seymour et al., 2008; Wright et al., 1995)

Table S3 related to table 2: Pancreatic transcription factor genes identified in mouse in which homozygous mutations have not been reported in patients with permanent neonatal diabetes. The phenotype observed in knock out (KO) mice is provided.

Supplemental References:

Ahlgren, U., Pfaff, S.L., Jessell, T.M., Edlund, T., and Edlund, H. (1997). Independent requirement for ISL1 in formation of pancreatic mesenchyme and islet cells. Nature *385*, 257-260.

Akazawa, H., Komuro, I., Sugitani, Y., Yazaki, Y., Nagai, R., and Noda, T. (2000). Targeted disruption of the homeobox transcription factor Bapx1 results in lethal skeletal dysplasia with asplenia and gastroduodenal malformation. Genes to cells : devoted to molecular & cellular mechanisms *5*, 499-513.

Al-Shawi, M., Al Mutair, A., Ellard, S., and Habeb, A.M. (2012). Variable phenotype in five patients with Wolcott-Rallison syndrome due to the same EIF2AK3 (c.1259delA) mutation. Journal of pediatric endocrinology & metabolism : JPEM, 1-4.

Ang, S.L., and Rossant, J. (1994). HNF-3 beta is essential for node and notochord formation in mouse development. Cell *78*, 561-574.

Artner, I., Blanchi, B., Raum, J.C., Guo, M., Kaneko, T., Cordes, S., Sieweke, M., and Stein, R. (2007). MafB is required for islet beta cell maturation. Proc Natl Acad Sci U S A *104*, 3853-3858.

Bi, W., Huang, W., Whitworth, D.J., Deng, J.M., Zhang, Z., Behringer, R.R., and de Crombrugghe, B. (2001). Haploinsufficiency of Sox9 results in defective cartilage primordia and premature skeletal mineralization. Proc Natl Acad Sci U S A *98*, 6698-6703.

Blanchi, B., Kelly, L.M., Viemari, J.C., Lafon, I., Burnet, H., Bevengut, M., Tillmanns, S., Daniel, L., Graf, T., Hilaire, G., *et al.* (2003). MafB deficiency causes defective respiratory rhythmogenesis and fatal central apnea at birth. Nat Neurosci *6*, 1091-1100.

Choo, D., Ward, J., Reece, A., Dou, H., Lin, Z., and Greinwald, J. (2006). Molecular mechanisms underlying inner ear patterning defects in kreisler mutants. Dev Biol *289*, 308-317.

Clotman, F., Lannoy, V.J., Reber, M., Cereghini, S., Cassiman, D., Jacquemin, P., Roskams, T., Rousseau, G.G., and Lemaigre, F.P. (2002). The onecut transcription factor HNF6 is required for normal development of the biliary tract. Development *129*, 1819-1828.

Coffinier, C., Gresh, L., Fiette, L., Tronche, F., Schutz, G., Babinet, C., Pontoglio, M., Yaniv, M., and Barra, J. (2002). Bile system morphogenesis defects and liver dysfunction upon targeted deletion of HNF1beta. Development *129*, 1829-1838.

Coffinier, C., Thepot, D., Babinet, C., Yaniv, M., and Barra, J. (1999). Essential role for the homeoprotein vHNF1/HNF1beta in visceral endoderm differentiation. Development *126*, 4785-4794.

Decker, K., Goldman, D.C., Grasch, C.L., and Sussel, L. (2006). Gata6 is an important regulator of mouse pancreas development. Dev Biol *298*, 415-429.

Ellard, S., Flanagan, S.E., Girard, C.A., Patch, A.M., Harries, L.W., Parrish, A., Edghill, E.L., Mackay, D.J., Proks, P., Shimomura, K., *et al.* (2007). Permanent neonatal diabetes caused by dominant, recessive, or compound heterozygous SUR1 mutations with opposite functional effects. Am J Hum Genet *81*, 375-382.

Garin, I., Edghill, E.L., Akerman, I., Rubio-Cabezas, O., Rica, I., Locke, J.M., Maestro, M.A., Alshaikh, A., Bundak, R., del Castillo, G., *et al.* (2010). Recessive mutations in the INS gene result in neonatal diabetes through reduced insulin biosynthesis. Proc Natl Acad Sci U S A *107*, 3105-3110.

Gierl, M.S., Karoulias, N., Wende, H., Strehle, M., and Birchmeier, C. (2006). The zinc-finger factor Insm1 (IA-1) is essential for the development of pancreatic beta cells and intestinal endocrine cells. Genes Dev *20*, 2465-2478.

Habeb, A.M., Al-Magamsi, M.S., Eid, I.M., Ali, M.I., Hattersley, A.T., Hussain, K., and Ellard, S. (2012). Incidence, genetics, and clinical phenotype of permanent neonatal diabetes mellitus in northwest Saudi Arabia. Pediatric diabetes *13*, 499-505.

Hallaq, H., Pinter, E., Enciso, J., McGrath, J., Zeiss, C., Brueckner, M., Madri, J., Jacobs, H.C., Wilson, C.M., Vasavada, H.*, et al.* (2004). A null mutation of Hhex results in abnormal cardiac development, defective vasculogenesis and elevated Vegfa levels. Development *131*, 5197-5209.

Haumaitre, C., Barbacci, E., Jenny, M., Ott, M.O., Gradwohl, G., and Cereghini, S. (2005). Lack of TCF2/vHNF1 in mice leads to pancreas agenesis. Proc Natl Acad Sci U S A *102*, 1490-1495.

Ishibashi, M., Ang, S.L., Shiota, K., Nakanishi, S., Kageyama, R., and Guillemot, F. (1995). Targeted disruption of mammalian hairy and Enhancer of split homolog-1 (HES-1) leads to up-regulation of neural helix-loop-helix factors, premature neurogenesis, and severe neural tube defects. Genes Dev *9*, 3136-3148.

Ito, T., Udaka, N., Yazawa, T., Okudela, K., Hayashi, H., Sudo, T., Guillemot, F., Kageyama, R., and Kitamura, H. (2000). Basic helix-loop-helix transcription factors regulate the neuroendocrine differentiation of fetal mouse pulmonary epithelium. Development *127*, 3913-3921.

Jacquemin, P., Durviaux, S.M., Jensen, J., Godfraind, C., Gradwohl, G., Guillemot, F., Madsen, O.D., Carmeliet, P., Dewerchin, M., Collen, D.*, et al.* (2000). Transcription factor hepatocyte nuclear factor 6 regulates pancreatic endocrine cell differentiation and controls expression of the proendocrine gene ngn3. Mol Cell Biol *20*, 4445-4454.

Jensen, J., Pedersen, E.E., Galante, P., Hald, J., Heller, R.S., Ishibashi, M., Kageyama, R., Guillemot, F., Serup, P., and Madsen, O.D. (2000). Control of endodermal endocrine development by Hes-1. Nat Genet *24*, 36-44.

Kaestner, K.H., Katz, J., Liu, Y., Drucker, D.J., and Schutz, G. (1999). Inactivation of the winged helix transcription factor HNF3alpha affects glucose homeostasis and islet glucagon gene expression in vivo. Genes Dev *13*, 495-504.

Kim, I., Saunders, T.L., and Morrison, S.J. (2007). Sox17 dependence distinguishes the transcriptional regulation of fetal from adult hematopoietic stem cells. Cell *130*, 470-483.

Martinez Barbera, J.P., Clements, M., Thomas, P., Rodriguez, T., Meloy, D., Kioussis, D., and Beddington, R.S. (2000). The homeobox gene Hex is required in definitive endodermal tissues for normal forebrain, liver and thyroid formation. Development *127*, 2433-2445.

Masui, T., Swift, G.H., Deering, T., Shen, C., Coats, W.S., Long, Q., Elsasser, H.P., Magnuson, M.A., and MacDonald, R.J. (2010). Replacement of Rbpj with Rbpjl in the PTF1 complex controls the final maturation of pancreatic acinar cells. Gastroenterology *139*, 270-280.

Mead, T.J., and Yutzey, K.E. (2012). Notch signaling and the developing skeleton. Adv Exp Med Biol *727*, 114-130.

Mihci, E., Turkkahraman, D., Ellard, S., Akcurin, S., and Bircan, I. (2012). Wolcott-Rallison syndrome due to a novel mutation (R491X) in EIF2AK3 gene. Journal of clinical research in pediatric endocrinology *4*, 101-103.

Molkentin, J.D., Lin, Q., Duncan, S.A., and Olson, E.N. (1997). Requirement of the transcription factor GATA4 for heart tube formation and ventral morphogenesis. Genes Dev *11*, 1061-1072.

Moriguchi, T., Hamada, M., Morito, N., Terunuma, T., Hasegawa, K., Zhang, C., Yokomizo, T., Esaki, R., Kuroda, E., Yoh, K.*, et al.* (2006). MafB is essential for renal development and F4/80 expression in macrophages. Mol Cell Biol *26*, 5715-5727.

Morrisey, E.E., Tang, Z., Sigrist, K., Lu, M.M., Jiang, F., Ip, H.S., and Parmacek, M.S. (1998). GATA6 regulates HNF4 and is required for differentiation of visceral endoderm in the mouse embryo. Genes Dev *12*, 3579-3590.

Nakhai, H., Siveke, J.T., Klein, B., Mendoza-Torres, L., Mazur, P.K., Algul, H., Radtke, F., Strobl, L., Zimber-Strobl, U., and Schmid, R.M. (2008). Conditional ablation of Notch signaling in pancreatic development. Development *135*, 2757-2765.

Oka, C., Nakano, T., Wakeham, A., de la Pompa, J.L., Mori, C., Sakai, T., Okazaki, S., Kawaichi, M., Shiota, K., Mak, T.W.*, et al.* (1995). Disruption of the mouse RBP-J kappa gene results in early embryonic death. Development *121*, 3291-3301.

Pfaff, S.L., Mendelsohn, M., Stewart, C.L., Edlund, T., and Jessell, T.M. (1996). Requirement for LIM homeobox gene Isl1 in motor neuron generation reveals a motor neuron-dependent step in interneuron differentiation. Cell *84*, 309-320.

Porter, J.R., Shaw, N.J., Barrett, T.G., Hattersley, A.T., Ellard, S., and Gloyn, A.L. (2005). Permanent neonatal diabetes in an Asian infant. The Journal of pediatrics *146*, 131-133.

Rosenbaum, J.N., Duggan, A., and Garcia-Anoveros, J. (2011). Insm1 promotes the transition of olfactory progenitors from apical and proliferative to basal, terminally dividing and neuronogenic. Neural development *6*, 6.

Rubio-Cabezas, O., Patch, A.M., Minton, J.A., Flanagan, S.E., Edghill, E.L., Hussain, K., Balafrej, A., Deeb, A., Buchanan, C.R., Jefferson, I.G., *et al.* (2009). Wolcott-Rallison syndrome is the most common genetic cause of permanent neonatal diabetes in consanguineous families. J Clin Endocrinol Metab *94*, 4162-4170.

Sander, M., Sussel, L., Conners, J., Scheel, D., Kalamaras, J., Dela Cruz, F., Schwitzgebel, V., Hayes-Jordan, A., and German, M. (2000). Homeobox gene Nkx6.1 lies downstream of Nkx2.2 in the major pathway of beta-cell formation in the pancreas. Development *127*, 5533-5540.

Seymour, P.A., Freude, K.K., Dubois, C.L., Shih, H.P., Patel, N.A., and Sander, M. (2008). A dosage-dependent requirement for Sox9 in pancreatic endocrine cell formation. Dev Biol *323*, 19-30.

Shaw-Smith, C., Flanagan, S.E., Patch, A.M., Grulich-Henn, J., Habeb, A.M., Hussain, K., Pomahacova, R., Matyka, K., Abdullah, M., Hattersley, A.T., *et al.* (2012). Recessive SLC19A2 mutations are a cause of neonatal diabetes mellitus in thiamine-responsive megaloblastic anaemia. Pediatric diabetes *13*, 314-321.

Shih, D.Q., Navas, M.A., Kuwajima, S., Duncan, S.A., and Stoffel, M. (1999). Impaired glucose homeostasis and neonatal mortality in hepatocyte nuclear factor 3alpha-deficient mice. Proc Natl Acad Sci U S A *96*, 10152-10157.

Sosa-Pineda, B., Chowdhury, K., Torres, M., Oliver, G., and Gruss, P. (1997). The Pax4 gene is essential for differentiation of insulin-producing beta cells in the mammalian pancreas. Nature *386*, 399-402.

Southwood, C., He, C., Garbern, J., Kamholz, J., Arroyo, E., and Gow, A. (2004). CNS myelin paranodes require Nkx6-2 homeoprotein transcriptional activity for normal structure. The Journal of neuroscience : the official journal of the Society for Neuroscience *24*, 11215-11225.

Spence, J.R., Lange, A.W., Lin, S.C., Kaestner, K.H., Lowy, A.M., Kim, I., Whitsett, J.A., and Wells, J.M. (2009). Sox17 regulates organ lineage segregation of ventral foregut progenitor cells. Developmental cell *17*, 62-74.

Sumazaki, R., Shiojiri, N., Isoyama, S., Masu, M., Keino-Masu, K., Osawa, M., Nakauchi, H., Kageyama, R., and Matsui, A. (2004). Conversion of biliary system to pancreatic tissue in Hes1-deficient mice. Nat Genet *36*, 83-87.

Sund, N.J., Vatamaniuk, M.Z., Casey, M., Ang, S.L., Magnuson, M.A., Stoffers, D.A., Matschinsky, F.M., and Kaestner, K.H. (2001). Tissue-specific deletion of Foxa2 in pancreatic beta cells results in hyperinsulinemic hypoglycemia. Genes Dev *15*, 1706-1715.

Turkkahraman, D., Bircan, I., Tribble, N.D., Akcurin, S., Ellard, S., and Gloyn, A.L. (2008). Permanent neonatal diabetes mellitus caused by a novel homozygous (T168A) glucokinase (GCK) mutation: initial response to oral sulphonylurea therapy. The Journal of pediatrics *153*, 122-126.

Watt, A.J., Zhao, R., Li, J., and Duncan, S.A. (2007). Development of the mammalian liver and ventral pancreas is dependent on GATA4. BMC Dev Biol *7*, 37.

Weinstein, D.C., Ruiz i Altaba, A., Chen, W.S., Hoodless, P., Prezioso, V.R., Jessell, T.M., and Darnell, J.E., Jr. (1994). The winged-helix transcription factor HNF-3 beta is required for notochord development in the mouse embryo. Cell *78*, 575-588.

Wright, E., Hargrave, M.R., Christiansen, J., Cooper, L., Kun, J., Evans, T., Gangadharan, U., Greenfield, A., and Koopman, P. (1995). The Sry-related gene Sox9 is expressed during chondrogenesis in mouse embryos. Nat Genet *9*, 15-20.

Xin, M., Davis, C.A., Molkentin, J.D., Lien, C.L., Duncan, S.A., Richardson, J.A., and Olson, E.N. (2006). A threshold of GATA4 and GATA6 expression is required for cardiovascular development. Proc Natl Acad Sci U S A *103*, 11189-11194.

Zhang, C., Moriguchi, T., Kajihara, M., Esaki, R., Harada, A., Shimohata, H., Oishi, H., Hamada, M., Morito, N., Hasegawa, K., *et al.* (2005). MafA is a key regulator of glucose-stimulated insulin secretion. Mol Cell Biol *25*, 4969-4976.

Zine, A., Aubert, A., Qiu, J., Therianos, S., Guillemot, F., Kageyama, R., and de Ribaupierre, F. (2001). Hes1 and Hes5 activities are required for the normal development of the hair cells in the mammalian inner ear. The Journal of neuroscience : the official journal of the Society for Neuroscience *21*, 4712-4720.
